# How Does Ambient Air Temperature Affect Diabetes Mortality in Tropical Cities?

**DOI:** 10.3390/ijerph14040385

**Published:** 2017-04-05

**Authors:** Xerxes T. Seposo, Tran Ngoc Dang, Yasushi Honda

**Affiliations:** 1 Graduate School of Comprehensive Human Sciences, University of Tsukuba, Tsukuba City 305-8577, Japan; ngocdangytcc@gmail.com; 2 Department of Environmental Health, Faculty of Public Health, University of Medicine and Pharmacy, Ho Chi Minh City 70000, Vietnam; 3 Faculty of Health and Sports Sciences, University of Tsukuba, Tsukuba City 305-8577, Japan; honda.yasushi.fn@u.tsukuba.ac.jp

**Keywords:** tropical cities, diabetes, temperature-mortality, low temperature effects, DLNM

## Abstract

Diabetes is well-known as one of the many chronic diseases that affect different age groups. Currently, most studies that evaluated the effects of temperature on diabetes mortality focused on temperate and subtropical settings, but no study has been conducted to assess the relationship in a tropical setting. We conducted the first multi-city study carried out in tropical cities, which evaluated the temperature–diabetes relationship. We collected daily diabetes mortality (ICD E10–E14) of four Philippine cities from 2006 to 2011. Same period meteorological data were obtained from the National Oceanic and Atmospheric Administration. We used a generalized additive model coupled with a distributed lag non-linear model (DLNM) in determining the relative risks. Results showed that both low and high temperatures pose greater risks among diabetics. Likewise, the study was able to observe the: (1) high risk brought about by low temperature, aside from the largely observed high risks by high temperature; and (2) protective effects in low temperature percentile. These results provide significant policy implications with strategies related to diabetes risk groups in relation to health service and care strategies.

## 1. Introduction

Diabetes mellitus (DM), commonly known as diabetes, is a group of metabolic diseases characterized by chronic hyperglycemia due to problems with insulin secretion, insulin action or both [1,2]. Globally, diabetes has been on the rise, and affects not just high-income countries, but has also spread and markedly increased among middle-income countries, which has claimed 1.5 million deaths [3]. In the Philippines, 4.6% of the general population have DM according to the 2003–2004 National Nutrition and Health Survey (NNHES) [4]. The increase in DM prevalence in the country through the years (see the Supplementary Materials Figure S1) may be attributed to multiple factors, which include but are not limited to sedentary lifestyle, variety of food consumption, and even climate change [5,6,7,8]. Effects of climate change, particularly how temperature affects human health, have been thoroughly studied with relatively similar patterns of increased risk in elderly and among patients with cardiovascular diseases in different cities and countries across the globe [9,10,11,12]. Similar studies have been done in the Philippines, wherein greater risks were observed among the elderly, cardiovascular-related diseases, and women [13,14]. In recent years, temperature effects on diabetes patients have been observed with increased susceptibility to both cold and hot temperatures [7]. Heat impairs both the thermoregulative and orthostatic responses at high temperatures, while apparent loss of efferent vasomotor control during diabetic neuropathy have been noted in cold periods [15,16]. However, most studies that explored the effects of temperature on diabetes mortality were mainly undertaken in temperate and subtropical cities [17,18,19,20,21,22,23,24,25,26,27]; to the best of our knowledge, no study has been carried out in a tropical setting. This is the first study which explored the effects of temperature on diabetes mortality in a multi-city tropical setting. Furthermore, this study quantified the risks associated with extreme low, moderate low, moderate high and extreme high temperatures.

## 2. Materials and Methods

### 2.1. Data

Daily DM mortality counts, with International Classifications of Diseases (ICD) 10 code E10–E14, were taken from the six-year daily all-cause mortality data from 2006 to 2011, provided by the Philippine National Statistics—National Statistics Office. Same period meteorological variables, such as temperature and dew point temperature, were obtained from the National Oceanic and Atmospheric Administration (NOAA). Temperature measurements in degrees Kelvin were converted to degrees Celsius, while relative humidity (*RHave*) was calculated using the following equation [28]:

(1) $$RHave=\;100-5\;\left(T_{ave}-T_{dew}\right)$$

where *T_ave_* is the average temperature and *T_dew_* is the dew point temperature, both in degrees Celsius.

We used low and high, instead of cold and hot, due to the subjectivity of definition with respect to the choice of words, particularly with the usage of “cold” in a tropical setting. Low and high temperatures were classified into four categories with respect to the reference temperature percentile: extreme low (25th temperature percentile vis-à-vis 1st temperature percentile), moderate low (25th temperature percentile vis-à-vis 10th temperature percentile), moderate high (75th temperature percentile vis-à-vis 90th temperature percentile), and extreme high (75th temperature percentile vis-à-vis 99th temperature percentile) [17,18]. We varied the maximum lags at 2, 7, 15 and 21 days to observe both the immediate and prolonged temperature effects on diabetes mortality [17].

Initial diagnostics included correlation analysis among the selected variables, univariate time series trends and simple linear regression (as seen in Supplementary Materials Figures S2–S4, respectively). Four tropical cities in the Philippines were included with respect to the completeness of both meteorological and mortality data: Manila City, Quezon City, Cebu City and Davao City.

### 2.2. Geographical Location

Philippines’ capital, Manila, is located in the National Capital Region, with a total population of 1,780,000. Quezon City, being the largest city in the same region, has 2,936,000 inhabitants as of 2015. Located in the middle part of the Philippines, in the Visayas group of islands, is Cebu City with a population of 923,000. In the southernmost cluster of islands in Mindanao, Davao City serves as the center of business and commerce with a population of 1,633,000 [29]. All four cities are situated in flat terrains, and have relatively similar temperature range. Year-round, the country experiences two distinct seasons, dry (January to May) and wet (June to December) [30].

### 2.3. Statistical Specifications

In estimating relative risks (RR), we used generalized additive quasi-Poisson model to quantify the association of daily diabetes mortality count and temperature [31]. Generalized additive models (GAM) are extensions of generalized linear models (GLM), which allow a more flexible usage of non-parametric functions such as penalized splines in controlling for the effects of time trends, and other covariates in the analysis [32].

Previous studies have shown that the association of temperature and diabetes mortality is nonlinear [17,18,19]. In order to address this nonlinearity, we used a distributed lag non-linear model (DLNM) to account for both the non-linear and delayed effects of temperature on diabetes mortality with a quasi-Poisson link to account for over-dispersion [9,31].

With the interchangeability due to high correlation of the temperature indicators, we used the daily average temperature over minimum and maximum temperatures, since it has better representation of the overall exposure throughout the day [17].

(2) $$\log\left[E\left(Y_{t}\right)\right]=\alpha +cb_{t,l}+s\left(time,k=y\times b\right)+s\left(RHave_{t},k=3\right)+DOW_{t}$$

where *Y_t_* is the number of diabetes deaths in day *t*; *E(Y_t_)* is the expected number of deaths in day *t*; α is the intercept; *k* is the degrees of freedom (df); *cb_t_*_,*l*_ is the cross-basis term in the respective temperature and lag dimensions with the best df combinations having the least Quasi-Akaike Information Criterion (QAIC); *s* is the fixed thin-plate regression spline with *k−1* df; *time* is a counter for each day of the observation period; *y* is the number of years in each city’s period; *b* is the selected df in modeling the *time* component; *RHave_t_* is the relative humidity with 3 df; and *DOW_t_* is the day of the week as an indicator variable.

We used 4 df for both lag and temperature dimensions in the cross-basis term, since it has the lowest QAIC value from among the various simulated combinations of both lag and temperature with each ranging from 4 to 20 df (see Supplementary Materials Table S1), respectively, which are supported by previous studies [9,10,11,12]. Seven degrees of freedom per year was used for time trends [10,11,13,33], while 3 df was used for *RHave*; both are in concurrence with previously published studies. We used different maximum lag, 0–2, 0–7, 0–15 and 0–21 days, to observe the immediate and delayed effects of temperature on diabetes mortality. After determining the non-linear exposure–response relationship, we examined the RRs with respect to the maximum lags and the temperature percentile definitions: extreme low, moderate low, moderate high, and extreme high.

All analyses were carried out using R programming (R Foundation for Statistical Computing, Vienna, Austria) through the “dlnm” and “mgcv” packages [34].

## 3. Results

Table 1 shows the descriptive statistics of diabetes mortality count per city, as well as the meteorological variables from 2006 to 2011. The mean and standard deviation of diabetes mortality for the respective cities are relatively the same, while non-diabetes mortality is varied, with Manila having most of the mortality (mean ≈ 49; ±SD ≈ 8). On the other hand, meteorological data are comparatively similar.

Table 2 shows the RRs in different temperature definitions (extreme low, moderate low, moderate high, and extreme high) and in different lag intervals (0–2, 0–7, 0–15 and 0–21 days). The highest RR across cities, lags, and temperature definitions, is recorded in Davao (RR = 3.87, 95% CI: 1.00–15.0). Aside from Davao’s extreme low temperature effects at lag 0–21, higher risks were observed in the extreme high temperatures among the cities. On the other hand, protective effects of temperature were observed in the extreme low and moderate low temperatures, exemplified by Cebu City, in lags 0–15 (extreme low (RR = 0.60, 95% CI: 0.13–2.75); moderate low (RR = 0.73, 95% CI: 0.29–1.83)), and 0–21 (extreme low (RR = 0.37, 95% CI: 0.06–2.15); moderate low (RR = 0.54, 95% CI: 0.19–1.58)). The pooled effects have shown greater effects in both moderate and extreme high temperatures.

**Figure 1:**
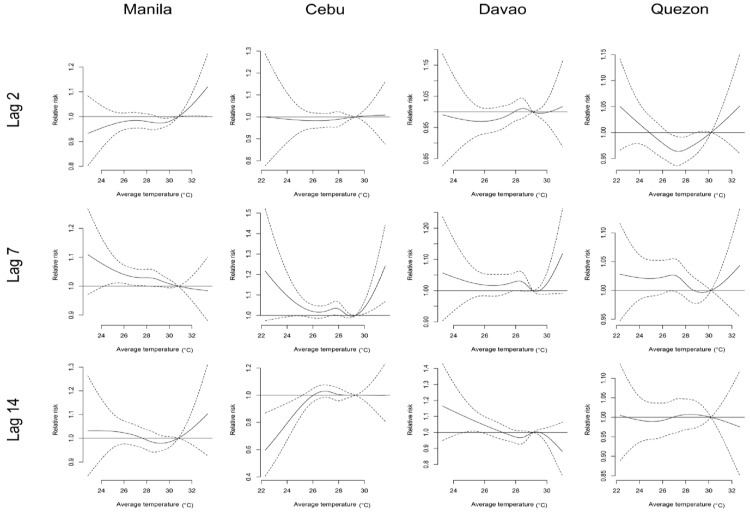
Diabetes mortality and temperature risk curves (centered at the 75th temperature percentile) with maximum lag 0–15 in various lag slices (at 2, 7 and 14) by city.

Figure 1 shows the slices of the exposure–response curve at lag interval 0–15 with the minimum mortality temperature (MMT) centered at the 75th temperature percentile. Evident low temperature effects can be observed at lag 7 in all cities. On the other hand, a protective effect can be observed in lag 14 of Cebu City.

**Figure 2:**
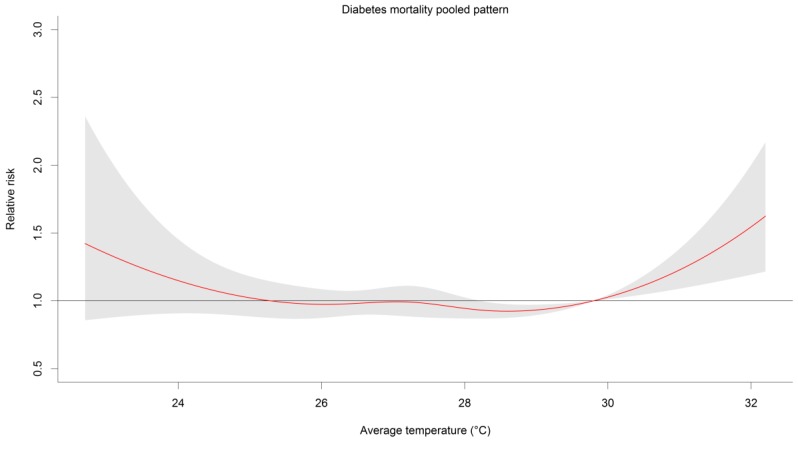
Pooled pattern of the risk curves of four tropical cities. Red solid line is the pooled pattern, while the gray areas are the 95% confidence interval.

Figure 2 shows the pooled pattern of the diabetes mortality. Evident high risks are observed in both low and high temperatures, with a steeper slope in the high temperature.

## 4. Discussion

In summary, the study determined the risks associated with low and high temperatures on diabetes, with relatively higher risks in extreme high temperatures; evident in the pooled pattern. Particularly, we have observed that: (1) tropical cities have increased risks even at lower temperature, aside from the commonly observed high risk due to high temperature; and (2) that low temperature exhibits protective effects against diabetes. Although the individual risk curves have shown inconsistent patterns, we believe that the findings of this first multi-city tropical study can provide insights in developing strategies specific to the diabetes risk population in relation to the temperature percentiles, unique to tropical cities and countries.

There have been numerous studies which have explored the effects of temperature on mortality, and have recorded similar patterns across different cities among various countries [10,11,12]. These patterns have been consistent not only in all-cause mortality, but also among specific mortality subgroups [13,31,35,36]. In this study, we have observed that the effects of temperature on diabetes are higher in extreme temperatures as seen in Table 2, which are consistent with literature [23,25,27]. The mechanism related to the increased risk in high temperatures among diabetics can be linked to the abnormalities of the thermoregulatory capacity caused by autonomic neuropathy [17,18,37]. Autonomic neuropathy is a potentially lethal diabetic complication, which affects multiple organ systems and of different clinical manifestations, with severe consequences such as hypoglycemia unawareness and cardiovascular dysfunction [38,39,40,41]. Furthermore, thermal stress intensifies the problems caused by autonomic neuropathy, by affecting the homeostasis, especially for cardiovascular and glycemia [42]. The thermal burden affects insulin absorption and various counter-regulatory hormones, which can affect both acute and chronic glycemia [17,42].

On the other hand, we have observed greater risks in either the extreme or moderate low temperatures, particularly in Cebu City (as seen in Table 2). At lag slice 7, in lag 0–15 as in Figure 1, most of the cities have greater risks in both the extreme and moderate low temperature percentiles. These results are similar to the studies in China [17,18,19]. The physiological mechanism of the increased risk of low temperatures on diabetes remains unclear. However, plausible associations can be linked towards too much exertion of the circulatory system leading to injured vasculature, which can be triggered by the extreme temperatures [18,43]. Likewise, other studies have noted that the increased levels of hemoglobin A1c (HbA1c) can be observed in winter periods [44,45,46]. HbA1c is associated with microvascular and macrovascular complications in diabetes [46]. The increased HbA1c level poses inherent risks, which can be further amplified by the injured vasculature.

On the other hand, protective effects were present in both extreme and moderate low temperatures, particularly in Cebu City, at lags 0–15 and 0–21, as seen in Table 2. The low temperature exposure may have therapeutic effect on diabetics, especially for type 2 patients [47,48,49]. This therapeutic effect can be attributed to the improved the glycemic control through improvements in insulin sensitivity brought about by cold temperature [42]. In order to prevent the decrease in the core body temperature, the body’s normal physiological response is to increase the rate of metabolic heat production induced by shivering and non-shivering thermogenesis [50]. This increase in metabolic energy production at low temperature further activates the brown adipose tissues, which oxidize triglycerides and glucose as fuel, thereby decreasing the glucose levels [51].

In a policy context, these results may prove to be useful in developing strategies focusing in the extreme temperature effects among diabetics, whereby, regardless of the lag in the city-specific risks, higher risks were observed in extreme temperatures. Diabetes patient care managers can use these results by managing accordingly the diabetic patients in relation to the temperature forecasts. For example, room temperature regulation can be endorsed during extreme temperature events. Similarly, there should be sufficient information dissemination of how both low and high temperatures affect diabetes patients even at a personal level management. Although this study has noted the protective effects of low temperature on diabetics, the causal pathway remains to be uncertain, whereby further research is warranted.

Although this study has brought forth insightful findings, we acknowledge the following limitations: (1) non-inclusion of pollution parameter; and (2) generalizability. We were not able to include the pollution parameter due to data unavailability. Nevertheless, effects estimates may still be similar studies regardless of the inclusion or non-inclusion of air pollution parameters based on previous studies [52,53,54]. The results of the study are unique to the study sites, and it would be difficult to generalize over the different parts of the Philippines due to geographic and socio-demographic orientations per area. However, results may be useful to a certain extent in areas of similar demographics.

## 5. Conclusions

Previous knowledge from studies carried out in temperate and subtropical settings have noted the risks in high temperatures with some studies indicating inclined risks even in low temperatures. This is the first multi-city, tropical-setting study carried out to investigate the effects of temperature on diabetes mortality. Results of this study have noted that the: (1) risks for diabetics not only exist in high temperatures, but also in low temperatures in a tropical setting; and (2) low temperatures may have protective effect on diabetes. Policymakers and health managers should take into account low temperature periods for diabetes management to equip the risk population of the necessary information with the goal of averting the risk.

## Figures and Tables

**Table 1:** City-specific descriptive statistics of the diabetes mortality and meteorological variables, 2006–2011.

**Variables**	**Manila**			**Quezon**		
	**Mean ± SD**	**Min**	**Max**	**Mean ± SD**	**Min**	**Max**
Diabetes mortality count	2.14 ± 1.47	0	9	2.76 ± 1.68	0	9
Maximum Temperature	31.5 ± 1.94	23.1	39.3	32 ± 2.21	24.4	38.4
Mean Temperature	28.5 ± 1.45	22.8	33.4	27.3 ± 1.56	22.3	32.8
Minimum Temperature	25.6 ± 1.45	18	31.3	23.4 ± 1.62	12.4	28.2
Relative Humidity	75.4 ± 8.31	42.8	96.9	80.0 ± 8.94	45.8	99.4
**Variables**	**Cebu**			**Davao**		
	**Mean ± SD**	**Min**	**Max**	**Mean ± SD**	**Min**	**Max**
Diabetes mortality count	1.46 ± 1.26	0	8	1.39 ± 1.20	0	7
Maximum Temperature	31.3 ± 1.63	24.8	40.5	31.9 ± 1.63	24	39.9
Mean Temperature	27.9 ± 1.24	22.3	31.6	28.3 ± 1.11	23.2	31
Minimum Temperature	24.8 ± 1.20	14.2	28	24.1 ± 0.78	16.2	27
Relative Humidity	85.1 ± 5.20	67.2	123.6	82.3 ± 4.57	66.7	97.5

SD: standard deviation.

**Table 2:** Cumulative RRs of cold and hot temperature effects on diabetes mortality along the various lag days.

City	Lag (days)	Extreme Low	Moderate Low	Moderate High	Extreme High
Manila					
	0–2	0.75 (0.43–1.29)	0.84 (0.60–1.16)	1.36 (1.03–1.80)	1.68 (1.03–2.74)
	0–7	1.09 (0.51–2.37)	1.06 (0.67–1.67)	1.54 (1.09–2.17)	2.11 (1.15–3.85)
	0–15	1.11 (0.40–3.07)	1.07 (0.58–1.97)	1.59 (1.04–2.42)	2.20 (1.04–4.65)
	0–21	1.01 (0.30–3.37)	1.02 (0.49–2.08)	1.52 (0.93–2.47)	2.04 (0.86–4.84)
Cebu					
	0–2	1.13 (0.53–2.44)	1.08 (0.68–1.71)	1.21 (0.95–1.54)	1.38 (0.87–2.19)
	0–7	1.77 (0.58–5.41)	1.40 (0.72–2.75)	1.57 (1.20–2.04)	2.32 (1.38–3.89)
	0–15	0.60 (0.13–2.75)	0.73 (0.29–1.83)	1.55 (1.16–2.08)	2.27 (1.27–4.04)
	0–21	0.37 (0.06–2.15)	0.54 (0.19–1.58)	1.54 (1.12–2.12)	2.20 (1.16–4.19)
Davao					
	0–2	1.02 (0.58–1.80)	1.01 (0.72–1.42)	0.98 (0.78–1.22)	1.02 (0.60–1.72)
	0–7	1.30 (0.54–3.09)	1.16 (0.69–1.96)	1.10 (0.82–1.48)	1.45 (0.71–2.98)
	0–15	1.44 (0.44–4.72)	1.25 (0.61–2.54)	1.04 (0.72–1.49)	1.14 (0.46–2.80)
	0–21	3.87 (1.00–15.0)	2.26 (1.01–5.08)	1.20 (0.82–1.76)	1.75 (0.67–4.57)
Quezon					
	0–2	1.34 (0.97–1.87)	1.20 (0.99–1.44)	1.16 (0.94–1.43)	1.29 (0.88–1.90)
	0–7	1.33 (0.81–2.18)	1.19 (0.90–1.57)	1.29 (0.99–1.68)	1.58 (0.98–2.57)
	0–15	1.75 (0.89–3.42)	1.38 (0.94–2.01)	1.32 (0.96–1.80)	1.65 (0.93–2.94)
	0–21	1.28 (0.58–2.84)	1.15 (0.73–1.81)	1.30 (0.92–1.84)	1.62 (0.85–3.07)
Pooled					
	0–2	1.03 (0.75–1.41)	1.01 (0.85–1.20)	1.24 (1.02–1.52)	1.35 (1.04–1.77)
	0–7	1.36 (0.96–1.93)	1.18 (0.98–1.41)	1.33 (1.09–1.62)	1.61 (1.21–2.15)
	0–15	1.39 (0.87–2.24)	1.19 (0.93–1.53)	1.28 (1.00–1.64)	1.55 (1.10–2.19)
	0–21	1.20 (0.54–2.69)	1.10 (0.71–1.71)	1.27 (0.95–1.71)	1.55 (1.06–2.29)

## Supplementary Materials

The following are available online at www.mdpi.com/1660-4601/14/4/385/s1. Figure S1: Diabetes prevalence from 1998–2013, Figure S2: Correlational relationship of minimum, average and maximum temperature, Figure S3: Time series trends of daily diabetes mortality per city from 2006–2011, Figure S4: Linear regression of diabetes mortality and average temperature per city, Table S1: Selection of the best combination of df for both temperature and lag dimensions.
