# Supplementary material for: How Does Ambient Air Temperature Affect Diabetes Mortality in Tropical Cities?
Source: Int J Environ Res Public Health. 2017 Apr 5;14(4):385. doi: 10.3390/ijerph14040385 (PMC5409586; doi:10.3390/ijerph14040385)
Supplement: Supplementary file 1 [file ijerph-14-00385-s001.pdf]

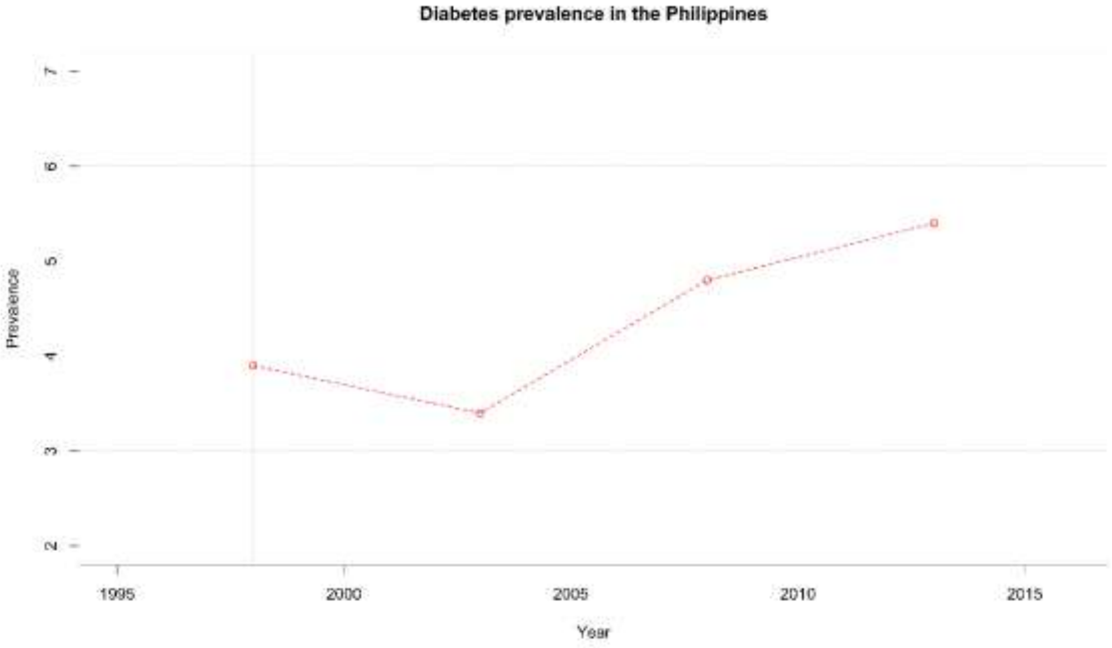

Figure S1. Diabetes prevalence from 1998-2013

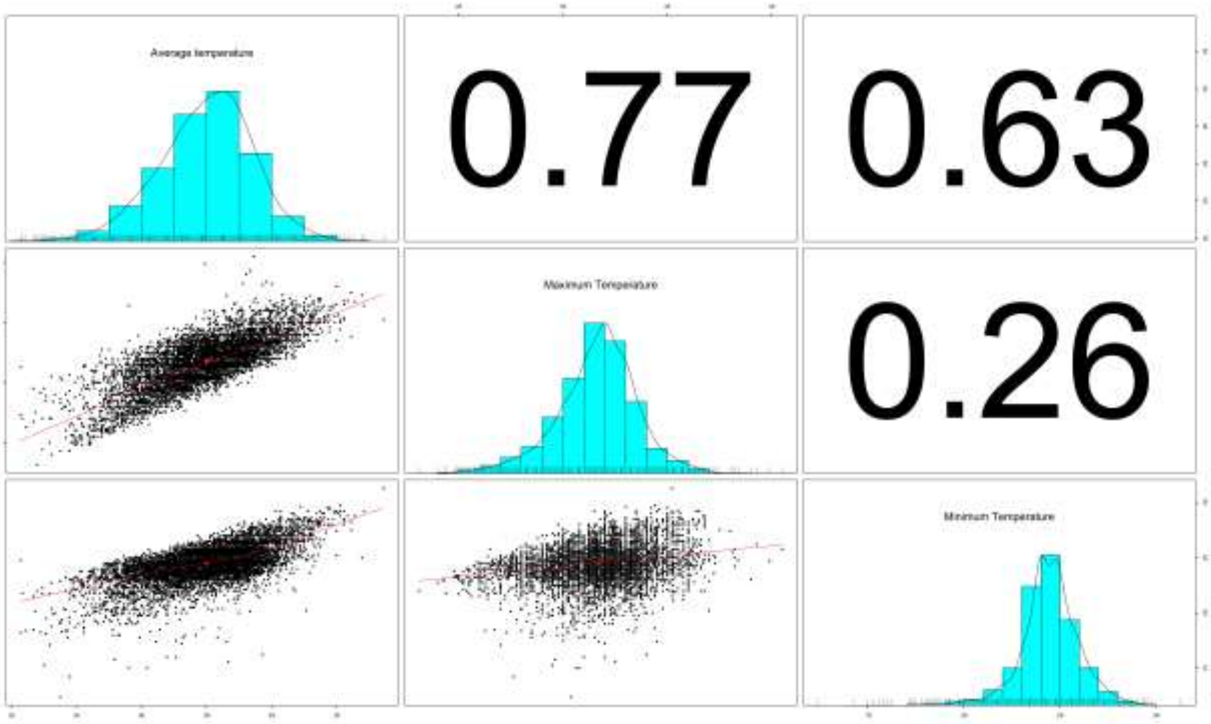

Figure S2. Correlational relationship of minimum, average and maximum temperature

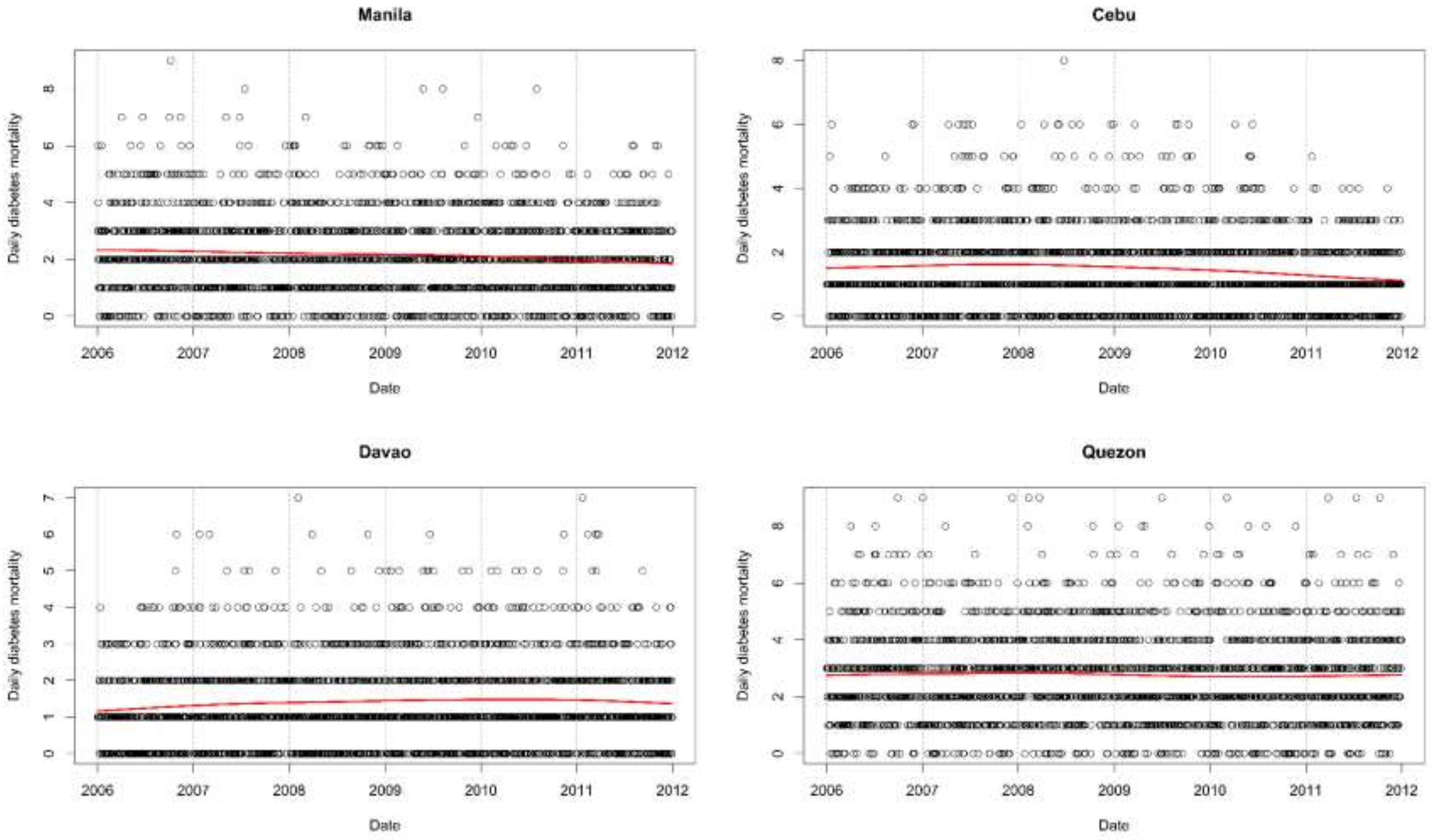

Figure S3. Time series trends of daily diabetes mortality per city from 2006-2011

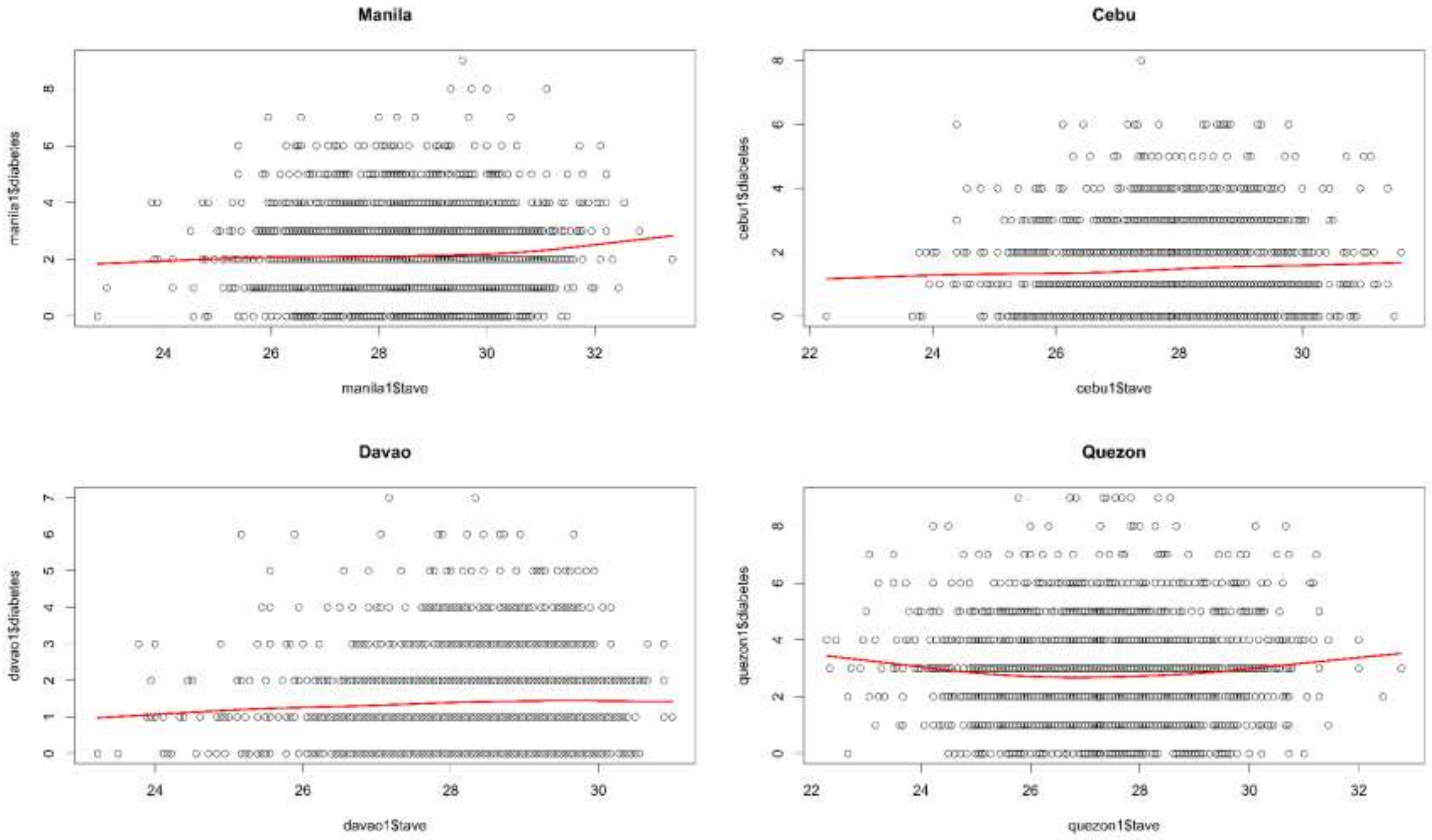

Figure S4. Linear regression of diabetes mortality and average temperature per city

Table S1. Selection of the best combination of df for both temperature and lag dimensions

Lag<br>Temperature	4 df	5 df	6 df	7 df	8 df	9 df	10 df	11 df	12 df	13 df	14 df	15 df	16 df	17 df	18 df	19 df	20 df
4 df	28886.75	28897.06	28910.18	28922.08	28940.78	28967.15	28979.91	28993.06	29009.52	29017.68	29028.39	29038.81	29056.49	29069.55	29078.56	29097.82	29116.7
5 df	28898.84	28911.82	28931.59	28948.3	28973.53	29002.59	29022.03	29038.71	29057.67	29071.67	29087.99	29105.49	29128.93	29145.96	29159.17	29183.46	29200.4
6 df	28898.65	28912.82	28934.1	28948.6	28983.05	29017.47	29043.71	29067.09	29089.77	29108.6	29130.41	29156.85	29180.16	29203.57	29224.12	29253.35	29267.89
7 df	28917.11	28932.74	28956.12	28974.8	29009.18	29050.07	29080.72	29108.89	29137.3	29158.76	29187.86	29218.97	29249.3	29280.73	29301.1	29329.08	29347.39
8 df	28925.25	28946.92	28975.82	28998.53	29039.75	29084.63	29116.36	29150.52	29180.05	29200.54	29235.97	29275.9	29303.3	29340.77	29364.84	29396.71	29423.27
9 df	28946.95	28974.12	29009.57	29034.3	29081.85	29123.6	29161	29199.76	29233.83	29259.28	29304.51	29345.95	29381.34	29425.56	29449.63	29486.01	29518.45
10 df	28951.09	28983.23	29015.42	29044.71	29100.31	29142.43	29180.9	29223.85	29261.62	29274.89	29323.23	29369.41	29411.99	29454.86	29479.22	29522.72	29556.26
11 df	28976.14	29014.12	29051.03	29084.2	29143.25	29193.6	29237.81	29288.1	29325.98	29344.53	29402.9	29452.94	29496.18	29545	29576.53	29626.63	29661.48
12 df	28974.91	29013.67	29050.68	29075.2	29134.1	29192.33	29238.14	29287.3	29331.91	29360.98	29419.15	29473.25	29523.05	29575.96	29614	29668.99	29705.52
13 df	28993.31	29038.32	29083.17	29119.78	29176.68	29242.74	29290.49	29342.25	29392.63	29421.53	29484.23	29542.17	29594.23	29655.38	29691.8	29749.11	29790.11
14 df	29010.96	29060.73	29109.81	29146.92	29203.15	29272.06	29328.13	29380.32	29432.6	29473.64	29535.34	29599.34	29656.32	29719.66	29766.69	29831.95	29882.68
15 df	29023.57	29074.82	29129.46	29163.97	29216.04	29293.7	29350.84	29411.51	29471.25	29515.17	29580.76	29649.85	29717.22	29785.67	29822.94	29885.53	29941.1
16 df	29044.22	29095.24	29156.47	29190.83	29252.87	29330.43	29395.13	29459.15	29524.63	29573.81	29648.89	29721.51	29784.1	29845.45	29900.6	29975.72	30029.58
17 df	29066.31	29127.57	29196.66	29240.1	29306.89	29393.71	29454.7	29524.77	29596.34	29651.17	29731.37	29813.86	29876.02	29958.96	30015.08	30086.49	30154.48
18 df	29077.97	29136.88	29209.99	29261.02	29327.11	29410.14	29484.77	29552.85	29624.58	29685.83	29766.11	29846.6	29913.64	29990.15	30054.24	30138.05	30184.82
19 df	29095.54	29161.01	29238.84	29289	29358.91	29448.65	29519.31	29600.84	29671.42	29738.67	29830.16	29919.42	29988.92	30071.29	30134.79	30211.44	30270.98
20 df	29114.8	29180.35	29261.68	29321.63	29386.48	29491.22	29566.19	29641.75	29719.68	29785.34	29885.37	29974.5	30053.93	30138.39	30221.36	30294.37	30354.23
